# *In vitro* assessment of the immunomodulatory effects of probiotic *Bacillus* strains on chicken PBMCs

**DOI:** 10.3389/fimmu.2024.1415009

**Published:** 2024-07-29

**Authors:** Filip Larsberg, Maximilian Sprechert, Deike Hesse, Clemens Falker-Gieske, Gunnar Loh, Gudrun A. Brockmann, Susanne Kreuzer-Redmer

**Affiliations:** ^1^ Albrecht Daniel Thaer-Institute, Animal Breeding and Molecular Genetics, Humboldt-Universität zu Berlin, Berlin, Germany; ^2^ Center for Animal Nutrition and Animal Welfare, Nutrigenomics, University of Veterinary Medicine Vienna, Vienna, Austria; ^3^ Department of Animal Sciences, Georg-August-Universität, Göttingen, Germany; ^4^ Center for Integrated Breeding Research, Georg-August-Universität, Göttingen, Germany; ^5^ Research, Development and Innovation, Nutrition and Care, Evonik Operations GmbH, Halle (Westfalen), Germany

**Keywords:** antimicrobial resistance, immune-modulating feed additives, probiotics, broiler chicken, *Bacillus* spp.

## Abstract

The beneficial effects of feeding probiotic *Bacillus subtilis* DSM 32315 (BS) and *Bacillus velezensis* CECT 5940 (BV) to chickens *in vivo* are well-documented, with potential immune modulation as a key mechanism. In this study, we investigated the direct interactions of chicken peripheral blood mononuclear cells (PBMCs) with BS or BV *in vitro* through whole transcriptome profiling and cytokine array analysis. Transcriptome profiling revealed 20 significantly differentially expressed genes (DEGs) in response to both *Bacillus* treatments, with twelve DEGs identified in BS-treated PBMCs and eight in BV-treated PBMCs. Pathway analysis using the Kyoto Encyclopedia of Genes and Genomes (KEGG) indicated significant regulation of immune-related pathways by both BS and BV. Notably, BS treatment upregulated genes associated with immune cell surface markers (CD4, CD25, CD28), anti-inflammatory cytokine interleukin-10 (IL-10), and C-C motif chemokine ligand 5 (CCL5), while downregulating the gene encoding pro-inflammatory IL-16. BV treatment similarly affected genes associated with immune cell surface markers, IL-16, and CCL5, with no impact on the gene encoding IL-10. Both treatments induced higher expression of the gene encoding the avian β-defensin 1 (AvBD1). The results of this *in vitro* study indicate an immunomodulatory effect of BS and BV in chicken PBMCs by regulating genes involved in anti-inflammatory, bacteriostatic, protective, and pro-inflammatory responses. Consequently, BS and BV may serve to augment the immune system’s capacity to defend against infection by modulating immune responses and cytokine expression. Thus, the administration of these probiotics holds promise for reducing reliance on antimicrobials in farming practices.

## 1 Introduction

Feed additives, such as probiotics (1), have the potential to improve the health of animals and humans and, thus, may help to prevent and reduce the usage of antimicrobials. Supplemental probiotics comprise mainly vital bacteria, fungi, and yeast that contribute to a healthy digestive system. Probiotics have varying and strain-specific beneficial effects on the host (2). These range from improving the intestinal epithelial barrier and excluding pathogenic bacteria to modulating the immune system (3). Immune-modulating probiotics, so-called immunobiotics, have been demonstrated to promote health by activating antigen-presenting cells (APCs) and modulating immune responses through the expression of immune-cell-specific genes (4). This potentially results in a more robust defense against infections and a balanced immune response.

APCs, including macrophages and dendritic cells, interact with probiotic bacteria by pattern recognition receptors (PRRs) such as Toll-like receptors on the surface of APCs. These receptors are capable of recognizing microbe-associated molecular patterns (MAMPs) which are present on probiotics. Activated APCs influence the differentiation of the immune response and regulate the production of pro- and anti-inflammatory cytokines. In mammals, probiotic bacteria were shown to modulate the balance between different T-helper cell types and their associated cytokines (5). Consequently, APCs can activate naive T cells and direct T-helper cell responses towards T-helper cell type 1 (Th1), Th2, Th17 or regulatory patterns involving regulatory T cells (Tregs) (6). In mammals, the Th1 immune response is primarily characterized by interferon-γ (IFN-γ) production, whereas the Th2 response is distinguished by the release of interleukin 4 (IL-4) and IL-5 (6). The Th17 response is characterized by the production of IL-17, whereas the induction of Tregs is primarily facilitated by transforming growth factor β (TGF-β) and associated with IL-10 or TGF-β production (6, 7). Additionally, probiotics have been demonstrated to facilitate the maturation of B cells into immunoglobulin A-producing plasma cells (6).

Moreover, probiotics were shown to enhance the production and secretion of host defense peptides (HDP), also known as antimicrobial peptides (AMPs) (8).

AMPs represent critical components linking the innate with the adaptive immune response (9). In chickens, AMPs include a total of 14 avian β-defensins (AvBDs) and four cathelicidins (CATHs) (10). AMPs are expressed in a wide range of tissues. They possess a broad spectrum of antimicrobial activities against various infectious agents, including bacteria, protozoa, viruses and fungi (10). In chickens, some of these AMPs, such as the leukocyte-derived AvBD1 and AvBD2, have been shown to have direct negative effects on microorganisms, including *Escherichia coli*, *Listeria monocytogenes*, and *Candida albicans* (11). Other experiments provided evidence for the induction of AMP production by feed additives such as butyrate, polyphenols, and peptidoglycan derived from *Lactobacillus rhamnosus* MLGA (12–14). Selected *Bacillus* species represent widely and often used probiotics, which are generally considered as biologically safe for the use in the poultry industry (15). Recently, we have reported T cell stimulation after *in vitro* exposure of chicken peripheral blood mononuclear cells (PBMCs) to two commercially available in-feed probiotics: *B. subtilis* DSM 32315 (BS) and *B. velezensis* CECT 5940 (BV, formerly known as *B. amyloliquefaciens* CECT 5940) (16). In the previous study, we examined the impact of both *Bacillus* strains on PBMCs, with a particular focus on the proportions of crucial T cell surface markers, including cluster of differentiation 4 (CD4), CD8, and CD25, which were quantified using flow cytometry.

To gain more insights into the mode of action of the *Bacillus* strains used, the current study aimed to assess the effects of vital BS or BV on chicken PBMCs on transcript and protein expression using RNA sequencing and a chicken cytokine array. Moreover, we ran Kyoto Encyclopedia of Genes and Genomes (KEGG)-based pathway analysis (17) to identify particularly affected and enriched pathways upon probiotic treatment.

This *in vitro* study enhances our understanding of how probiotics influence immune cells, paving the way for targeted administration of probiotics in animal feed to enhance chicken health and combat infectious diseases. It also establishes a foundation for future research exploring optimal probiotic formulations and application strategies in poultry farming, aimed at reducing the need for subtherapeutic antimicrobials and mitigating risks associated with microbial resistance.

## 2 Materials and methods

### 2.1 Animals, housing, feeding, and tissue collection

In total, seven four- to six-week-old broiler chickens of the commercial breed Cobb500 (Cobb Germany Avimex GmbH) were used. Broiler chickens were fed a starter diet from day 1 to day 14 post hatch, and a grower diet afterwards (H. Wilhelm Schaumann GmbH, Pinneberg, Germany). The animals were fed *ad libitum* and water was always available. For experiments, the birds were stunned and decapitated. The blood was sampled in sodium citrate (Na-citrate) pre-filled polystyrene tubes (VACUETTE®, Greiner Bio-One, Kremsmünster, Austria). The study was approved by the local state office of occupational health and technical safety “Landesamt für Gesundheit und Soziales Berlin” (LaGeSo Reg. T 0151/19, T-HU-07/21).

### 2.2 PBMC isolation and culture

PBMCs were isolated using combined dextran-ficoll isolation as described previously (18). In brief, blood was diluted with Dulbecco’s phosphate-buffered saline (DPBS, Gibco™, ThermoFisher Scientific, Waltham, Massachusetts, USA) containing 2 mM ethylenediaminetetraacetic acid (EDTA, Carl Roth, Karlsruhe, Germany) 1:1, mixed with 3% dextran (Carl Roth) in a ratio of 1:0.4, and centrifuged at 50 x g for 20 min. Thereafter, the upper phase was collected, layered onto an equal volume of Histopaque-1077 (Sigma-Aldrich, St. Louis, Missouri, USA), and centrifuged at 900 x g for 30 min. The PBMCs at the interphase were collected, washed twice with DPBS/EDTA in a new 50 ml tube, and centrifuged at 400 x g for 10 min. Isolated PBMCs were adjusted to 5x10**^6^** cells/ml vital cells using Tali™ image-based cytometer (ThermoFisher Scientific) with propidium iodide (PI, ThermoFisher Scientific) as a viability marker. PBMCs were cultured in RPMI 1640 medium (Gibco™, ThermoFisher Scientific) with 10% chicken serum (Gibco™, ThermoFisher Scientific) and 1% penicillin (10000 U/ml)-streptomycin (10000 µg/ml) (Pen/Strep, Gibco™, ThermoFisher Scientific) at 41°C with 5% CO_2_. The next day, further experiments, as for example the co-culture of PBMCs with *Bacillus* strains, were performed.

### 2.3 Bacterial strains and culture

RPMI 1640 medium was inoculated with either *B. subtilis* DSM 32315 (BS) or *B. velezensis* CECT 5940 (BV). Bacteria were cultured overnight at 37°C and 120 rpm. To define the actual colony forming units per milliliter (cfu/ml), the bacterial growth (OD600) was measured on a Tecan Infinite® M200 Pro plate reader with Magellan™ v. 7.1 software (Tecan Group AG, Männedorf, Switzerland). Bacterial cultures were plated on tryptic-soy agar (TSA, Carl Roth) on petri dishes (Greiner Bio-One, Frickenhausen, Germany) and counted the next day.

### 2.4 Probiotic treatment/co-culture

Co-culture experiments were conducted using probiotic bacterial strains and 1x10^6^ PBMCs with either BS or BV in a ratio of 1:3 (PBMC: *Bacillus*). This ratio was found to be optimal in previous experiments (16). The experiments were carried out in 24-well plates (Eppendorf, Hamburg, Germany) for 24 h. All experiments were performed in RPMI 1640 medium containing 10% chicken serum without Pen/Strep at 41°C with 5% CO_2_. For all co-culture experiments, concanavalin A (conA, Vector Laboratories, Newark, California, USA) was used as a positive control for cell proliferation. PBMCs were treated in a concentration of 10 µg/ml conA. After 24 h, cells were harvested and centrifuged at 400 x g for 10 min. The co-culture supernatants were transferred to cryogenic vials (Corning®, VWR International, Radnor, Pennsylvania, USA). The co-culture supernatants and the cell pellets were stored at -80°C for chicken cytokine array analyses and RNA isolation, respectively.

### 2.5 Cytokine measurement

The Quantibody® chicken cytokine array Q1 (RayBiotech, Peachtree Corners, GA, USA) was used to detect a selection of cytokines and chemokines (IL-6, IL-10, IL-12, IL-16, IL-21, CCL5), which are related to inflammatory processes, in the supernatants of the co-culture of chicken PBMCs with BS or BV (see 2.4). Sample preparation as well as data acquisition and analysis were performed by RayBiotech from co-culture supernatants which were sent to the company on dry ice.

### 2.6 RNA isolation

Total RNA was isolated from PBMCs from six different chickens (biological replicates) of pooled experimental (technical) duplicates from each chicken after treatment with either BS, BV or conA or untreated samples (medium) according to the manufacturer’s total RNA isolation protocol (NucleoSpin RNA Plus XS, Macherey-Nagel, Düren, Germany). Quantification of RNA was performed using a NanoDrop instrument (ND-1000, ThermoFisher Scientific). Quality was further controlled by a fluorescence-based quantification method and fragment length analysis by CeGaT GmbH. For total RNA sequencing (RNA-Seq), the integrity number (RIN) of the RNA had to be >4.

### 2.7 RNA sequencing and bioinformatics

Total RNA sequencing using a 100-base paired-end approach was carried out on an Illumina NovaSeq 6000 platform (Illumina, San Diego, CA, USA) by CeGat GmbH (Tübingen, Germany). Libraries were prepared from 10 ng of total RNA using the SMART-Seq Stranded Kit (Takara Bio Inc., Kusatsu, Shiga, Japan). Demultiplexing of sequencing reads of different samples was performed with Illumina bcl2fastq (version 2.20). Adaptors were trimmed with Skewer (version 0.2.2). Trimmed raw reads were aligned to GRCg6a (GCA_000002315.5) using STAR (version 2.7.3). The quality of the FASTQ files was analyzed with FastQC (version 0.11.5). Differential gene expression between treatment and control groups was analyzed using DESeq2 (version 1.24.0) in R (version 3.6.1). The raw counts derived from the mapping contain the number of reads that map to each geneID. Based on these numbers the normalized read counts were calculated. In the first step of the normalization, DESeq2 calculates a fictive “reference sample” which is defined as the geometric mean for each gene across all samples regardless of group affiliation. The counts for each gene and sample are afterwards divided by this reference value. In the next step, the size factor is estimated for each sample by calculating the median of these ratios. To get the normalized read counts for each gene and sample, the raw counts were divided by the sample’s size factor. This normalization accounts for different library sizes and for biases. Genes with less than two reads over all samples were removed. Using the normalized read counts, the Log_2_ fold change (FC) was calculated and tested for statistical significance by Wald test. Benjamini-Hochberg correction was used to correct for multiple testing. Genes with a Log_2_ FC > |1.5| and adjusted p-value (padj) < 0.05 were considered as significantly differentially expressed.

In addition to the global analyses, we specifically analyzed the normalized read counts of immune cell receptor genes which were obtained by DESeq2 with a padj > 0.05, such as *CD4*, *CD8*, *CD25*, and *CD28*. The related cell surface receptors were shown to be affected on T cells in chicken PBMCs upon treatment with probiotic BS and BV in a previous study (16). Moreover, we investigated the normalized read counts of cytokines with a padj > 0.05 and compared them with the results of the protein concentrations in co-culture supernatants obtained by the cytokine array. The normality of the data was assessed using the Shapiro-Wilk test. Depending on the distribution, a paired t-test or Wilcoxon rank-sum test was performed to compare the effects of probiotic treatment with the untreated control. Scatter dot plots showing the mean with standard deviation (SD) were chosen for graphical representation of the gene expression data and protein concentrations in co-culture supernatants. The graphical representations and analyses were conducted using GraphPad Prism 8.0.2 (GraphPad Software, San Diego, CA, USA). Statistical significance was considered at p < 0.05, with the following symbols indicating the level of significance: * = p < 0.05, ** = p < 0.01. Statistical tendencies were noted at p < 0.1, indicated by +. The RNA-sequencing data used in this study can be found in the Gene Expression Omnibus (GEO) under accession no. GSE272225.

### 2.8 Gene set enrichment analysis

Gene cluster comparisons and visualizations were achieved with the R package clusterProfiler (version 4.8.1) (19). Gene symbols were converted to ensemble IDs with the clusterProfiler Biological Id Translator (bitr). KEGG pathway analysis was done with enrichKEGG (settings: pvalueCutoff = 1, pAdjustMethod = “BH”, minGSSize = 10, maxGSSize = 500, qvalueCutoff = 0.25, use_internal_data = FALSE). Genes with p < 0.05 and an absolute Log_2_ FC > 0.5 were used as input. Plots were drawn with the dotplot function.

## 3 Results

### 3.1 Differentially expressed genes and differentially regulated pathways between BS- or BV-treated and untreated PBMCs

**Figure 1:**
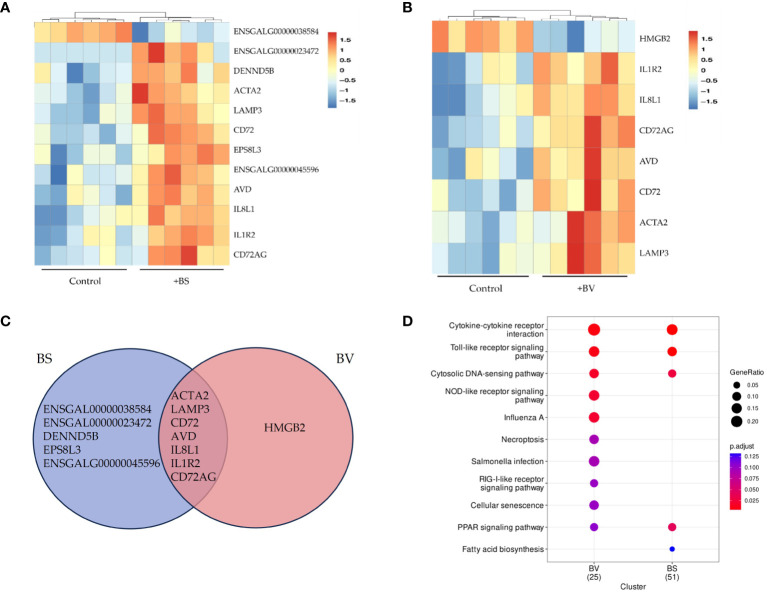
Differentially expressed genes in chicken PBMCs after exposure to vital *B*. *subtilis* DSM 32315 (BS) and *B*. *velezensis* CECT 5940 (BV). **(A)** BS and **(B)** BV: Heatmap of the differentially expressed genes between BS or BV treatment and the control. The hierarchical clustering shows two main groups. Red tiles represent up-regulated genes with adjusted p-values (padj) below 0.05. Blue tiles represent down-regulated genes with padj below 0.05. Darker colors correspond to higher values (range between -1.5 and 1.5). **(C)** Venn diagram showing the number of overlapping differentially expressed genes between BS-treated and BV-treated PBMCs. **(D)** KEGG enrichment for differentially expressed genes (Log_2_ FC > 0.5, p < 0.05) in two clusters, namely BS and BV. The size of each circle shows the gene ratio, representing the percentage of significant genes over the total genes in a given pathway. Different colors of each circle indicate the adjusted p-value (p.adjust) as false discovery rate (FDR). The significantly enriched KEGG pathways are shown. Data represent the results of six biological replicates after treatment with BS or BV.

The comparative analysis of PBMCs cultured as a negative control and PBMCs treated with the probiotics BS or BV revealed twelve differentially regulated genes that were affected by BS treatment and eight genes after BV treatment. Seven of the significantly differentially expressed genes (DEGs) were overlapping between both groups of probiotic treatments (**Figures 1A–C**).

In BS-treated PBMCs, eleven DEGs (Log_2_ FC > 1.5, padj < 0.05) were up-regulated (*IL1R2*, *DENND5B*, ENSGALG00000045596*, IL8L1, CD72, CD72AG, AVD, ACTA2, LAMP3*, ENSGALG00000023472*, EPS8L3*) and one was downregulated (ENSGALG00000038584) (**Figures 1A, C**). Hierarchical clustering clearly revealed two distinct groups one for the untreated control samples and one for the BS-treated PBMCs (**Figure 1A**). KEGG pathway analysis was performed with 252 differentially expressed genes (Log_2_ FC > 0.5, p < 0.05) in BS-treated PBMCs. Four significantly enriched KEGG pathways (padj < 0.05) were identified, namely cytokine-cytokine receptor interaction, toll-like receptor (TLR) signaling pathway, cytosolic DNA-sensing pathway, and peroxisome proliferator-activated receptor (PPAR) signaling pathway (**Figure 1D** and **Supplementary Table 1**). Among those, the cytokine-cytokine receptor interaction pathway displayed the highest level of enrichment.

For PBMCs treated with BV, eight DEGs (Log_2_ FC > 1.5, padj < 0.05) were found of which seven were upregulated (*IL1R2, IL8L1, CD72, CD72AG, AVD, ACTA2, LAMP3*) and one was downregulated (*HMGB2*) (**Figures 1B, C**). KEGG pathway analysis of 154 differentially expressed genes (Log_2_ FC > 0.5, p < 0.05) identified five pathways being significantly enriched (padj < 0.05). These pathways were cytokine-cytokine receptor interaction, TLR signaling, cytosolic DNA-sensing, nucleotide-binding oligomerization domain-like receptor signaling, and influenza A (**Figure 1D** and **Supplementary Table 2**).

### 3.2 Effects of BS and BV on immune cell receptor gene expression in PBMCs

**Figure 2:**
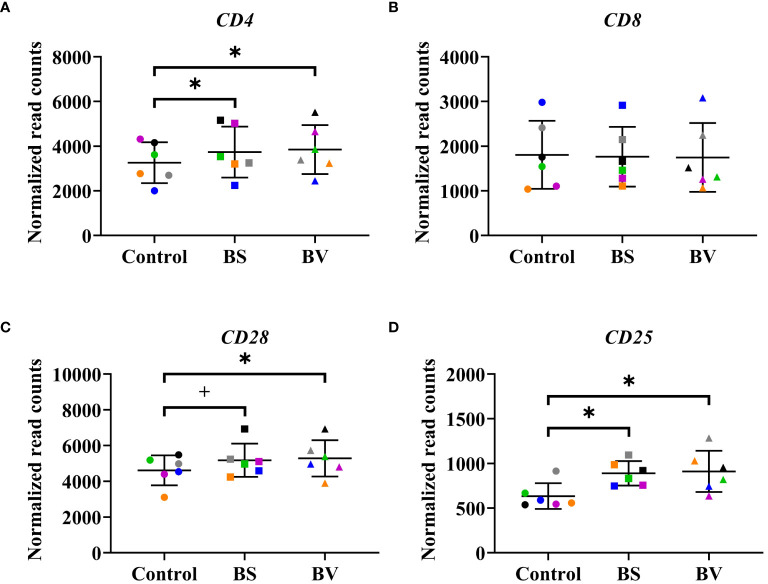
Influence of vital *B*. *subtilis* DSM 32315 (BS) and *B*. *velezensis* CECT 5940 (BV) on the expression of immune cell surface marker genes in chicken PBMCs. **(A)** *CD4* receptor gene; **(B)** *CD8* receptor gene; **(C)** *CD28* receptor gene; **(D)** *CD25* receptor gene. Data represent normalized read counts of six biological replicates after treatment with BS or BV. Results are presented as scatter dot plots showing the mean with SD. Individual values represent the mean of the technical replicates per biological replicate and are shown as circles (Control), squares (BS), and triangles (BV). The same color refers to the same individual for control and BS or BV treatment. A paired t-test (*CD4*, *CD*8, *CD28*) or a Wilcoxon rank-sum test (*CD25*) was performed. Significance is shown as +, p < 0.1; *, p < 0.05.

In a previous study, we reported the activation and proliferation of distinct T cell populations as a response of chicken PBMCs to BS and BV. We evaluated the effects of BS or BV on CD4+ T-helper cells, CD4+CD25+ activated T-helper cells, CD8+ cytotoxic T cells, CD8+CD25+ activated cytotoxic T cells, and CD28+ T cells by means of the proportions of the cell surface markers by flow cytometry and observed an elevated T cell immune response in chicken PBMCs (16). In the present study, we investigated the expression of the related immune cell surface marker genes after treatment of PBMCs with BS or BV (**Figure 2**). Additionally, we analyzed the expression of transcription factors that were previously identified as being responsible for the differentiation of Th1 and Th2 cells. Specifically, we examined the gene expression of GATA-binding protein 3 (*GATA3*) and T-box transcription factor 21 (*TBX21*) (**Supplementary Figure 1**).

On gene expression level, the treatment of PBMCs with BS revealed an increase of the expression level of the CD4 receptor gene (*CD4*) by a mean value of 476 counts (p < 0.05, **Figure 2A**) compared to the untreated control. Furthermore, the expression level of the immune cell activation marker genes *CD28* and *CD25* were elevated by a mean value of 564.7 (p < 0.1, **Figure 2C**) and 254.9 counts (p < 0.05, **Figure 2D**), respectively, in the presence of BS compared to the negative control. The expression level of the *CD8* receptor gene remained unchanged (**Figure 2B**). The expression of *GATA3* and *TBX21* did not change after treatment with BS (**Supplementary Figure 1**).

The treatment of PBMCs with BV also increased the expression level of the *CD4* receptor gene by 588.8 counts (p < 0.05, **Figure 2A**). Moreover, the expression levels of the activation marker genes *CD28* and *CD25* were elevated by a mean value of 670.1 (p < 0.05, **Figure 2C**) and 276.6 counts (p < 0.05, **Figure 2D**), respectively, compared to the untreated negative control. The expression level of the *CD8* receptor gene remained also unchanged (**Figure 2B**). Similar to the treatment with BS, the expression of *GATA3* and *TBX21* remained unchanged after treatment with BV (**Supplementary Figure 1**).

These results corroborate our previous findings obtained by flow cytometry, which indicated an elevated number of CD4+ T-helper cells, CD4+CD25+ activated T-helper cells, and CD28+ T cells. In contrast, the unaltered gene expression level of the *CD8* receptor gene (**Figure 2B**) does not align with the elevated number of CD8+ cytotoxic T cells and CD8+CD25+ activated cytotoxic T cells observed in the preceding flow cytometric assessments following treatment with BS. However, it is essential to acknowledge that the receptor genes are not exclusive to T cells, but also express the associated receptors on other immune cell types.

### 3.3 Expression of genes encoding cytokines in BS- and BV-treated PBMCs

**Figure 3:**
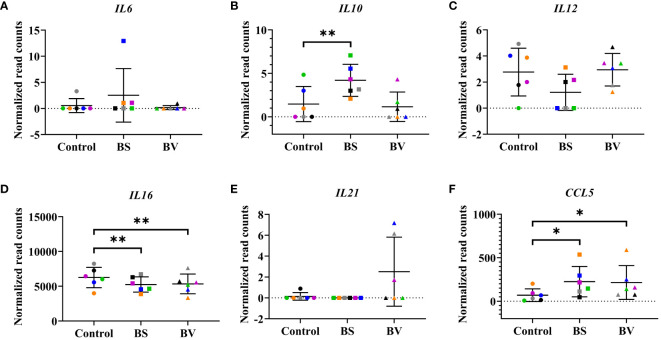
Influence of vital *B*. *subtilis* DSM 32315 (BS) and *B*. *velezensis* CECT 5940 (BV) on the profile of cytokine genes in chicken PBMCs. **(A)** *IL6*; **(B)** *IL10*; **(C)** *IL12*; **(D)** *IL16*; **(E)** *IL21*; **(F)** *CCL5*. Data represent normalized read counts of six biological replicates after treatment with BS or BV. Results are presented as scatter dot plots showing the mean with SD. Individual values represent the mean of the technical replicates per biological replicate and are shown as circles (Control), squares (BS), and triangles (BV). The same color refers to the same individual for control and BS or BV treatment. A paired t-test (BS: *IL10*, *IL12*, *IL16*, *CCL5*; BV: *IL12*, *IL16*) or a Wilcoxon rank-sum test (BS: *IL6*, *IL21*; BV: *IL6*, *IL10*, *IL21*, *CCL5*) was performed. Significance is shown as *, p < 0.05; **, p < 0.01.

**Figure 4:**
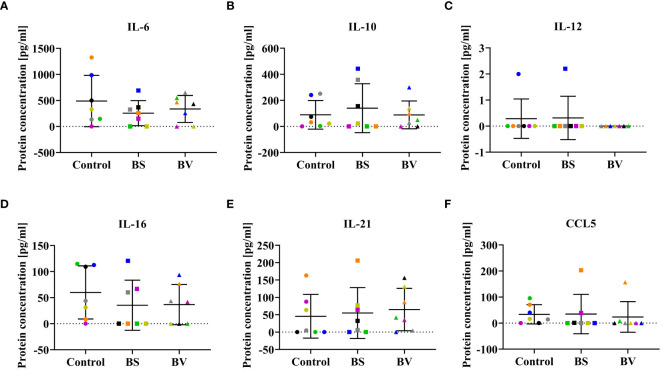
Influence of vital *B*. *subtilis* DSM 32315 (BS) and *B*. *velezensis* CECT 5940 (BV) on the cytokine profile in co-culture supernatants. Cytokine concentrations of **(A)** IL-6, **(B)** IL-10, **(C)** IL-12, **(D)** IL-16, **(E)** IL-21, and **(F)** CCL5. Data represent normalized read counts of seven biological replicates after treatment with BS or BV. Results are presented as scatter dot plots showing the mean with SD. Individual values represent the mean of the technical replicates per biological replicate and are shown as circles (Control), squares (BS), and triangles (BV). The same color refers to the same individual for control and BS or BV treatment. A paired t-test (BS: IL-6; BV: IL-6, IL-16) or a Wilcoxon rank-sum test (BS: IL-10, IL-12, IL-16, IL-21, CCL5; BV: IL-10, IL-12, IL-21, CCL5) was performed.

In order to assess the effects of BS and BV on PBMCs, we further evaluated the cytokine expression levels (**Figures 3A–F**, **4A–F**). After treatment with BS, we observed increased expression levels of the anti-inflammatory cytokine gene *IL10* by a mean value of 2.728 (p < 0.01, **Figure 3B**) and the C-C motif chemokine ligand 5 (*CCL5*) by 156.1 counts (p < 0.05, **Figure 3F**), but reduced expression level of the pro-inflammatory cytokine gene *IL16* by 1004 counts (p < 0.01, **Figure 3D**) compared to the untreated controls. The pro-inflammatory cytokine genes *IL12* (**Figure 3C**) and *IL6* (**Figure 3A**), and the regulatory cytokine gene *IL21* (**Figure 3E**) remained unaffected by BS. In contrast, the pro-inflammatory cytokine genes *IL1B* (p < 0.05, **Supplementary Figure 2A**) and *IL8* (p < 0.05, **Supplementary Figure 2B**) were increased after treatment with BS by 39.35 and 122.2 counts, respectively. The expression level of the regulatory cytokine gene *TGFB1* decreased by 181.4 counts (p < 0.05, **Supplementary Figure 3A**), while the mean expression level of *TGFB2* exhibited a slight increase, although this result was not statistically significant (**Supplementary Figure 3B**).

In PBMCs treated with BV, we observed an increase in gene expression of *CCL5* by a mean value of 146.1 counts (p < 0.05, **Figure 3F**) and a decrease in expression of *IL16* by 920.2 counts (p < 0.01, **Figure 3D**) compared to the untreated controls, similar to what was observed in BS-treated PBMCs. The treatment of PBMCs with BV had no effect on the expression levels of the pro-inflammatory cytokine genes *IL6* (**Figure 3A**) and *IL12* (**Figure 3C**), the anti-inflammatory cytokine gene *IL10* (**Figure 3B**), and the regulatory cytokine gene *IL21* (**Figure 3E**). Additionally, following treatment with BV, the expression of the pro-inflammatory cytokine genes *IL1B* and *IL8* was found to be significantly increased by 38.76 (p < 0.05, (**Supplementary Figure 2A**) and 126.7 counts (p < 0.05, **Supplementary Figure 2B**), respectively. The expression level of *TGFB1* significantly decreased by 214.0 counts (p < 0.05, **Supplementary Figure 3A**), while the expression level of *TGFB2* did not change compared to the control (**Supplementary Figure 3B**).

### 3.4 Cytokine concentrations in co-culture supernatants of PBMCs treated with BS and BV

Since cytokines were expected to be released by different immune cell populations during the co-culture of probiotics and PBMCs, we investigated the concentrations of potentially functional cytokines. In our experiments, cytokine concentrations in co-culture supernatants of PBMCs treated with BS or BV were not significantly altered. No differences in concentrations of pro-inflammatory IL-6, IL-12, and IL-16, anti-inflammatory IL-10, regulatory IL-21, and CCL5 were detected after treatment with either BS or BV compared to the untreated control (**Figure 4**).

### 3.5 Effects of BS and BV on gene expression of antimicrobial peptides in PBMCs

**Figure 5:**
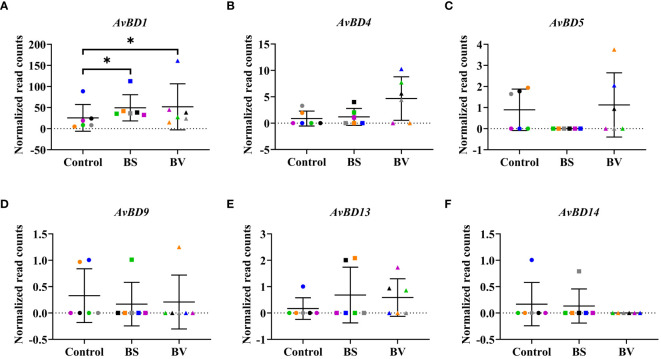
Expression of avian β-defensin genes in chicken PBMCs after treatment with vital *B*. *subtilis* DSM 32315 (BS) and *B*. *velezensis* CECT 5940 (BV). Expression levels of **(A)** *AvBD1*, **(B)** *AvBD4*, **(C)** *AvBD5*, **(D)** *AvBD9*, **(E)** *AvBD13*, and **(F)** *AvBD14*. Data represent normalized read counts of six biological replicates after treatment with BS or BV. Results are presented as scatter dot plots showing the mean with SD. Individual values represent the mean of the technical replicates per biological replicate and are shown as circles (Control), squares (BS), and triangles (BV). The same color refers to the same individual for control and BS or BV treatment. A Wilcoxon rank-sum test was performed. Significance is shown as *, p < 0.05.

In addition to cytokine gene expression, we analyzed the expression levels of antimicrobial peptides (AMPs) which influence the expression of cytokines and *vice versa*. We investigated the changes in the gene expression of the avian β-defensins *AvBD1*, *AvBD4, AvBD5, AvBD9, AvBD13*, and *AvBD14* (**Figure 5**) as well as the cathelicidins *CATH1, CATH2, CATH3*, and *CATHB1* (**Supplementary Figure 4**) in PBMCs after treatment with BS or BV compared to the untreated controls.

The effects of BS and BV on the expression of the investigated AMPs in PBMCs were similar. After BS treatment of PBMCs, we observed a higher expression level of *AvBD1* by a mean value of 23.93 counts (p < 0.05, **Figure 5A**). The β-defensin genes *AvBD4*, *AvBD5*, *AvBD9*, *AvBD13*, and *AvBD14* remained unaffected after treatment with BS (**Figure 5**). After BV treatment, the expression level of *AvBD1* also increased in a similar magnitude as after BS treatment, by a mean value of 26.27 counts (p < 0.05, **Figure 5A**), and the β-defensin genes *AvBD4*, *AvBD5*, *AvBD9*, *AvBD13*, and *AvBD14* did not change (**Figure 5**).

The expression of the second major group of AMPs, the cathelicidins, in particular *CATH1, CATH2, CATH3*, and *CATHB1*, did not change after treatment with BS or BV compared to the untreated controls (**Supplementary Figures 4A–D**).

## 4 Discussion

We detected twelve differentially expressed genes (DEGs) in PBMCs treated with BS and eight in PBMCs treated with BV compared to the untreated controls. The seven overlapping DEGs are *IL1R2, IL8L1, CD72, CD72AG*, *AVD*, *ACTA2*, and *LAMP3*. These genes encode the IL-1 receptor 2 (IL-1R2), IL-8-like 1 (IL-8L1), CD72 molecule (CD72), CD72 antigen (CD72AG, also known as CD72 molecule like 1 (CD72L1)), avidin (AVD), actin-α 2 (ACTA2), and lysosomal-associated protein 3 (LAMP3). Remarkably, all of those genes are involved in immune regulation. To identify the enriched pathways for the regulated genes upon BS and BV treatment of PBMCs, KEGG pathway analysis was performed. Immune-related pathways, namely cytokine-cytokine receptor interaction, Toll-like receptor (TLR) signaling pathway, and cytosolic DNA-sensing pathway were significantly enriched. These results suggest strong interactions of innate as well as adaptive immune cells in response to an activating or modulating stimulus, which in case of BS or BV could involve secreted molecules or surface molecules, such as peptidoglycans (20). Moreover, the results indicate the involvement of specific pattern recognition receptors, namely TLRs or cytosolic nucleotide sensors such as nucleotide-binding oligomerization domain like (NOD-like) receptors, which respond to different components of microorganisms by inducing innate immune responses. The involvement of TLRs and NOD-like receptors underlines the pro-inflammatory response upon treatment of PBMCs with BS and BV and suggests a strengthened immune response capacity. In a recent *in vivo* study, similar pathways, including the TLR signaling pathway, NOD-like receptor signaling pathway, and cytokine-cytokine receptor interaction, were significantly enriched in the cecum of chickens that were fed *B. subtilis* throughout the experimental trial (day 1 to day 28 of age) and simultaneously challenged with *Eimeria tenella* on day 21 of age compared to chickens fed a normal diet (without the probiotic) and challenged with *E. tenella* on day 21 (21).

IL-1R2, whose gene expression was regulated by both bacterial strains, is a profound mediator in inflammatory and immune responses to various disorders (22). In mammals, IL-1R2 is expressed in monocytes and macrophages, neutrophils, and B cells (23). There, the expression of IL-1R2 is elevated upon exposure to anti-inflammatory or immunosuppressive agents, such as prostaglandins (24) or aspirin (25). The functional role of IL-1R2 in chickens is not well understood. Similar to humans, IL-2R1 has been shown to specifically bind IL-1β in chickens, thereby inhibiting its activity (26, 27). In this study, higher *IL1R2* gene expression was induced in chicken PBMCs after treatment with both bacterial strains, BS and BV. This suggests the presence of anti-inflammatory agents secreted by the bacteria or an overexpression of IL-1β. These results are further consistent with another study reporting the upregulation of the *IL1R2* gene following T cell receptor (TCR) stimulation of regulatory T cells (Tregs) and our previous results which indicated elevated counts of CD4+CD25+ T cells, which were suggested to represent a T cell population with regulatory functions in chickens, following treatment of chicken PBMCs with BS and BV (16, 28).

The mammalian cytokine IL-8 and the chicken homologous IL-8L1 are members of the CXC family of chemokines, acting mainly on neutrophils as well as on T and B cells (29). In our study, the expression of the *IL8* gene was significantly elevated upon treatment with both bacterial strains. In a recent study, human PBMCs were co-cultured with *Streptococcus thermophilus* 285, which also resulted in elevated IL-8 protein expression. The authors suggested that IL-8 alone may indicate inflammation, but in the context of other upregulated anti-inflammatory cytokines and mediators found in their study, upregulation of IL-8 may be interpreted as a requirement for the initial stimulatory effect of *S. thermophilus* 285 to activate the immune response by initiating innate immunit*y*, which in turn influences adaptive immune responses (30). Thus, the initial pro-inflammatory immune response observed in our study, characterized by the upregulation of genes like *IL1R2* and *IL8L1* in PBMCs treated with BS and BV, may serve as a precursor to the involvement of Tregs, as suggested in previous research, particularly following treatment with BS (16).

The mammalian B cell differentiation antigen CD72 and the most homologous chicken protein CD72AG are type II transmembrane proteins of the C-type lectin family and are mainly expressed on the B cell linage except for plasma cells (31). There, the CD72 protein functions as a B cell antigen receptor (BCR)-mediated signaling inhibitor (32). Thus, higher expression of the *CD72* gene after treatment with BS and BV indicates BCR-mediated signaling inhibition. Accordingly, we could not observe an effect on the number of B cells after treatment with BS or BV by flow cytometry in a previous study (16).

AVD is a tetrameric protein with antimicrobial properties found in egg whites of all oviparous vertebrates. Each AVD monomer can reversibly bind biotin (vitamin H) with high affinity and specificity. The binding makes biotin unavailable for microorganisms and prevents their proliferation. Therefore, AVD can be considered bacteriostatic (33). In our experiments, it was higher expressed in *Bacillus*-treated PBMCs compared to the untreated controls. The higher expression of the *AVD* gene in *Bacillus*-treated PBMCs in our study suggests a probiotic bacteriostatic effect of BS and BV.

The expression of ACTA2 is largely restricted to smooth muscle cells, pericytes, and myofibroblasts. However, ACTA2 was reported to be upregulated in PBMCs from dogs with heart failure (34) or in children immunized with inactivated influenza vaccine (35). In our study, the gene encoding ACTA2 was upregulated following treatment with both *Bacillus* strains, indicating increased actin cytoskeleton and integrin signaling as it was suggested in another study (35).

The lysosomal-associated protein 3 (LAMP3) is a transmembrane lysosomal glycoprotein and a reliable activation marker for basophils (36). In humans, LAMP3 is enriched in dendritic cells and T cells (37), which is in line with the elevated T cell immune response in our previous study (16).

The study suggests that both probiotics induce an anti-inflammatory and bacteriostatic effect characterized by an increase in the gene expression of *IL1R2* and *AVD*, and the involvement of T cells, particularly Tregs. The increased expression of pro-inflammatory cytokine genes such as *IL1B* and *IL8* indicates significant interactions between innate and adaptive immune cells in response to an activating or modulating stimulus, involving specific pattern recognition receptors, including TLRs and NOD-like receptors.

In addition to investigate gene regulation globally after treatment of PBMCs with BS or BV, we specifically examined the expression of immune cell surface marker genes, which are associated with the previously suggested elevated T cell immune response in chicken PBMCs upon treatment with BS or BV (16). In the previous study, elevated levels of CD4, CD8, CD25, and CD28 cell surface markers were found using flow cytometry (16). These results were underlined through RNA sequencing analysis, which showed higher expression levels of the immune cell surface marker genes *CD4*, *CD25*, and *CD28* after treatment with the tested *Bacillus* strains. However, the analysis of differential gene expression of those genes did not result in significant differences on a global gene expression level. The observed effects on the gene expression of cell surface markers of individual cell populations may be low due to the heterogeneity of the PBMC population. PBMCs contain different populations of immune cells, which may hide specific effects on individual cell populations.

Furthermore, it is important to note that the higher gene expression of those cell surface markers does not necessarily reflect the previously reported elevated T cell immune response by flow cytometry measurement of important T cell receptors. The aforementioned genes, specifically *CD4*, *CD8*, and *CD25*, also encode the cell surface markers of other cell types present in the PBMC population, including natural killer (NK) cells and monocytes. However, these are found to a lesser extent compared to, e.g. T cells, in the peripheral blood (38). Although also other immune cell populations within PBMCs express similar cell surface receptors, the upregulation of the surface receptor marker genes and the T cell-associated *CCL5*, as well as the higher expression of the B cell proliferation inhibitor gene *CD72* after PBMC treatment with BS and BV, may indicate a potential stimulation of the T cell immune response, but not the B cell response.

In contrast to previous findings on protein levels obtained by flow cytometry, the expression of the cell surface marker gene *CD8* was not elevated. This could be a result of different cell types expressing CD8 receptors, including NK cells (38). One hypothesis for the unchanged expression of *CD8* upon treatment with BS or BV could also involve a downregulation of the *CD8* gene in response to a regulatory immune reaction involving a CD4+CD25+ T-helper cell population with regulatory properties that possess similar functions to those of mammalian Tregs, as previously suggested (16, 39). This hypothesis is supported by the increased expression level of the regulatory cytokine gene *IL10* which points towards an involvement of Tregs. However, we could not detect differences in the concentrations of the IL-10 protein, a typical Treg type cytokine, in the co-culture supernatants. It has to be noted that the CD4+CD25+ T cell population also includes other subsets than just Tregs. Recently, a chicken ortholog of the mammalian Foxp3 was identified (40). In the future, the production of chicken Foxp3 antibodies could give better opportunities for studying Treg subsets.

Other cytokines involved in a Treg driven immune response are TGF-β and IL-2. While the *TGFB2* gene was only slightly but not significantly increased after treatment with BS in this study, the *TGFB1* gene was significantly downregulated after treatment of PBMCs with both *Bacillus* strains. This is in line with the findings of a recent study which could show that, while gene expression levels of the cytokines IL-10 and IL-2 in chicken T cell subsets generally resemble their mammalian counterparts, the expression of the cytokine TGF-β was not associated with other typical Treg proteins (40). Correspondingly, we could only detect a low expression level of the *IL2* gene, whose expression is normally suppressed by Tregs (41), suggesting a very low expression (data not shown). Those results suggest an increase of Tregs after chicken PBMC treatment with BS or BV. Another study has identified TGF-β as a potential substitute marker for chicken Tregs in Marek’s disease, defining a Treg subset that is largely distinct from CD4+CD25+ T cells (42). The authors of this study have proposed that TGF-β may serve as a marker for peripherally induced chicken Tregs. The expression of TGF-β was investigated using an anti-TGF-beta1,2,3 antibody. The utilization of an antibody against TGF-β in forthcoming experiments may facilitate a more precise delineation of the Treg population posited in the present study.

To further exclude a Th1 or Th2 immune response, we analyzed the gene expressions of the GATA-binding protein 3 (*GATA3*), encoding the transcription factor responsible for the differentiation of Th2 cells (43, 44), and the T-box transcription factor 21 (*TBX21*), encoding a ‘master regulator’ of cell-mediated immunity. The latter is capable of controlling the expression of genes encoding effector molecules, such as CD4+ Th1 cells and CD8+ cytotoxic T cells (45, 46). While the mean of the *GATA3* expression slightly, but not significantly, increased, the *TBX21* expression did not change. However, this result does not allow for suggestions or the exclusion of a Th1 or Th2 immune response.

The expression of genes encoding the pro-inflammatory and regulatory cytokines IL-6, IL-12, and IL-21 remained unchanged after PBMC treatment with either BS or BV. In contrast, the gene encoding the pro-inflammatory cytokine IL-16 was downregulated, while the gene encoding CCL5 was upregulated after treatment with both bacterial strains. Furthermore, treatment with BS led to elevated expression of the gene encoding IL-10. IL-16 was initially described as a lymphocyte chemoattractant factor mainly synthesized by T cells, in particular CD4+ and CD8+ T cells, in response to antigens, mitogens, histamine, and serotonin (47). Correspondingly, its expression was elevated after treatment of chicken PBMCs with conA in the current study (data not shown). Thus, the downregulation of the *IL16* gene upon treatment suggests an anti-inflammatory effect of both bacterial strains. This result is consistent with the increased expression of the cytokine genes *IL10* and *CCL5*, indicating T cell involvement, specifically that of Tregs. In this study, the gene expression of *IL6*, *IL10*, *IL12*, *IL16*, *IL21*, and *CCL5* did not correlate with the abundance of these cytokines in the supernatants of the co-culture experiments. This finding suggests varying degrees of correlation between gene expression and secreted proteins, which was also reported in a previous study (48).

The expression of AvBD and CATH genes, which encode important AMPs, did not change after treatment with BS and BV, except for the *AvBD1* gene. In a recent study, *in ovo* administration of ABD1 significantly protected chicks from early mortality caused by experimental yolk sac infection with avian pathogenic *E. coli*, suggesting its immunomodulatory and anti-infection activity (49). Furthermore, a protective and antimicrobial effect of AvBD1 was reported against *E. coli*, *L. monocytogenes*, and *C. albicans* in chicken leukocytes (11). In our study it remains to be elucidated if the elevated expression levels of *AvBD1* in PBMCs upon treatment with BS or BV offers protection to experimental infections. TLR-2, which commonly recognizes the pattern molecules of gram-positive bacteria (50) did not seem to be involved in the changes in *AvBD1* expression as the expression of *TLR2* did not change after treatment with both, BS and BV (data not shown). Therefore, additional other factors as short-chain organic acids produced by the bacteria may be involved in changes in the expression of AMPs as it was shown previously in the jejunum and cecum of chickens (51).

## 5 Conclusions

Our results indicate an anti-inflammatory and bacteriostatic effect of both probiotics, BS and BV. Evidence is provided by increased expression of *IL1R2* and *AVD*, while at the same time the expression of pro-inflammatory cytokine genes such as *IL1B* and *IL8* was also increased. Additionally, we observed altered expression of immune cell surface marker genes, including *CD4*, *CD28*, and *CD25* after treatment with both tested probiotic *Bacillus* strains in our cell culture system. Furthermore, we observed a modulation of the expression patterns of genes encoding pro-inflammatory and anti-inflammatory cytokines, as well as protective compounds such as IL-1β, IL-8, IL-10, IL-1R2, AVD, and AvBD1 by both *Bacillus* strains. Moreover, we identified significantly enriched KEGG pathways comprising innate and adaptive immune responses. The findings of this *in vitro* study provide a foundation for future research and evidence that *B. subtilis* DSM 32315 and *B. velezensis* CECT 5940 could potentially enhance the immune system’s ability to defend against infection by modulating immune responses and cytokine expression. This could help to prevent and reduce the use of antimicrobials in chicken farming.

## Data availability statement

The datasets presented in this study can be found in online repositories. The names of the repository/repositories and accession number(s) can be found here: https://www.ncbi.nlm.nih.gov/geo, accession number: GSE272225.

## Ethics statement

The animal study was approved by local state office of occupational health and technical safety “Landesamt für Gesundheit und Soziales Berlin” (LaGeSo Reg. T 0151/19, T-HU-07/21). The study was conducted in accordance with the local legislation and institutional requirements.

## Author contributions

FL: Data curation, Formal analysis, Investigation, Visualization, Writing – original draft, Writing – review & editing. MS: Investigation, Writing – review & editing. DH: Supervision, Writing – review & editing. CF-G: Formal analysis, Visualization, Writing – review & editing. GL: Writing – review & editing. GB: Conceptualization, Supervision, Writing – review & editing. SK-R: Conceptualization, Supervision, Writing – review & editing.
