# Supplementary material for: In vitro assessment of the immunomodulatory effects of probiotic Bacillus strains on chicken PBMCs
Source: Front Immunol. 2024 Jul 29;15:1415009. doi: 10.3389/fimmu.2024.1415009 (PMC11320415; doi:10.3389/fimmu.2024.1415009)
Supplement: Supplementary file 1 [file DataSheet_1.docx]

Supplementary Material

*In vitro* assessment of the immunomodulatory effects of probiotic *Bacillus* strains on chicken PBMCs

Filip Larsberg^1,5^, Maximilian Sprechert^1^, Deike Hesse^1^, Clemens Falker-Gieske^2,3^, Gunnar Loh^4^, Gudrun A. Brockmann^1^, Susanne Kreuzer-Redmer^5*^

*** Correspondence:** Corresponding Author: susanne.kreuzer-redmer@vetmeduni.ac.at

# Supplementary Figures

#
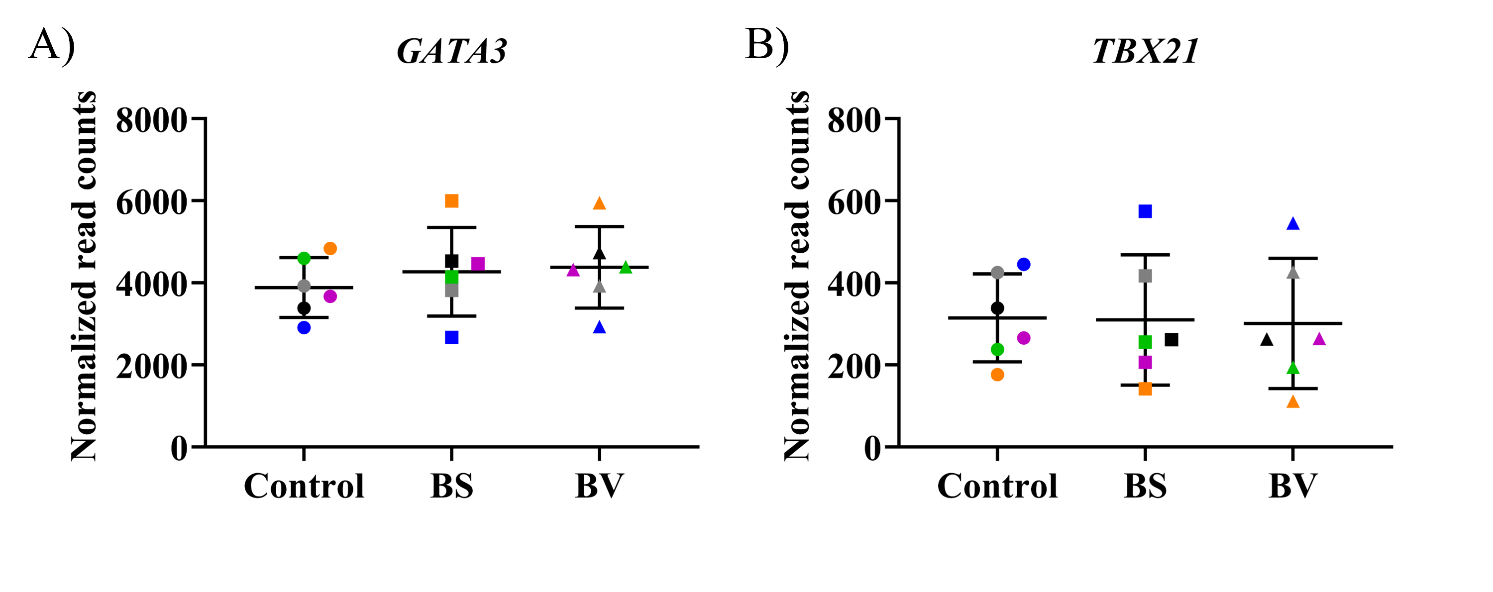


**Supplementary Figure 1.** Influence of vital *B. subtilis* DSM 32315 (BS) and *B. velezensis* CECT 5940 (BV) on the expression of major transcription factor genes (A) *GATA3* and (B) *TBX21* in chicken PBMCs. Data represent normalized read counts of six biological replicates after treatment with BS or BV. Results are presented as scatter dot plots showing the mean with SD. Individual values represent the mean of the technical replicates per biological replicate and are shown as circles (Control), squares (BS), and triangles (BV). The same colour refers to the same individual for control and BS or BV treatment. A paired t-test was performed.


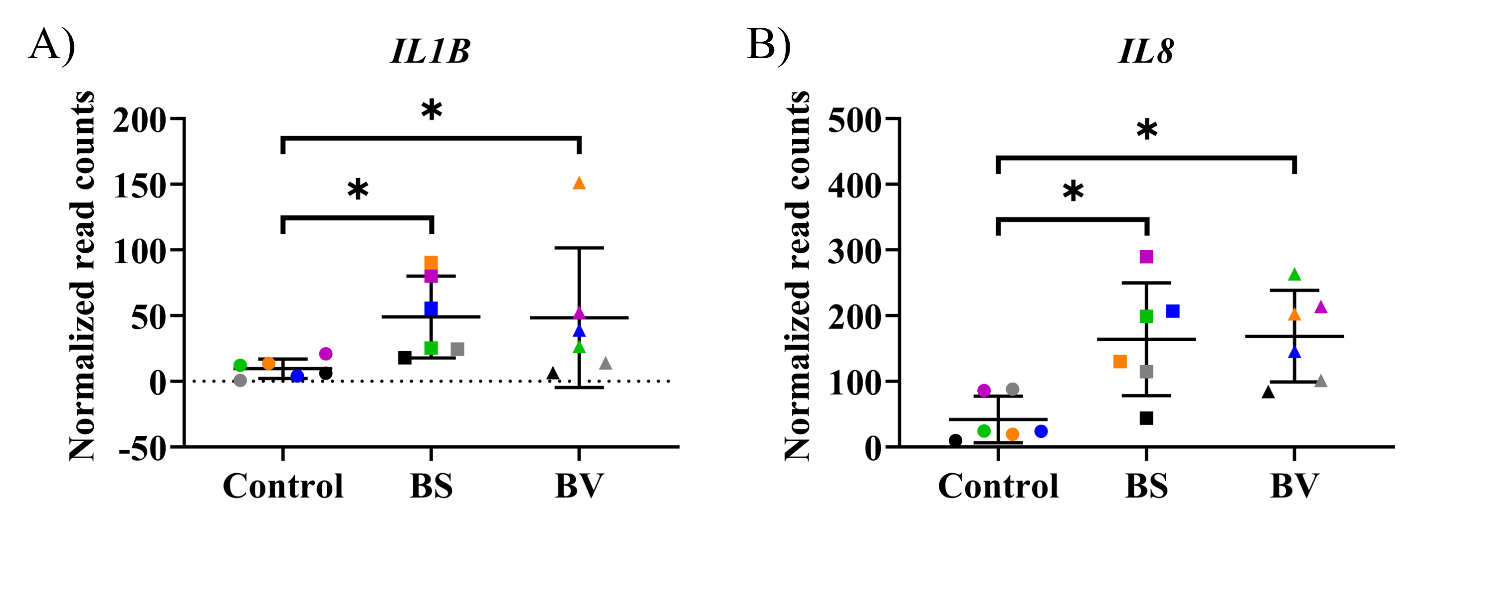


**Supplementary Figure 2.** Influence of vital *B. subtilis* DSM 32315 (BS) and *B. velezensis* CECT 5940 (BV) on the expression of pro-inflammatory cytokine genes (A) *IL1B* and (B) *IL8* in chicken PBMCs. Data represent normalized read counts of six biological replicates after treatment with BS or BV. Results are presented as scatter dot plots showing the mean with SD. Individual values represent the mean of the technical replicates per biological replicate and are shown as circles (Control), squares (BS), and triangles (BV). The same colour refers to the same individual for control and BS or BV treatment. A paired t-test (BS: *IL1B*) and a Wilcoxon rank-sum test (BS: *IL8*; BV: *IL1B*, *IL8*) was performed. Significance is shown as *, p < 0.05.


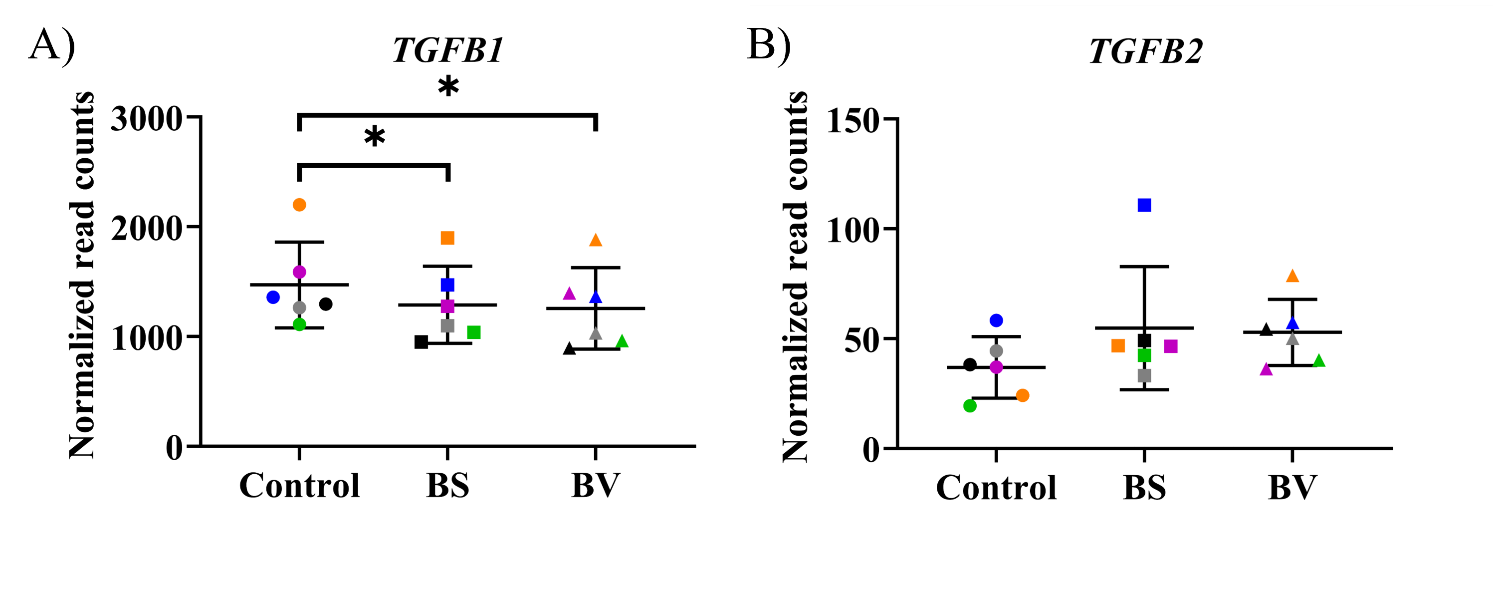


**Supplementary Figure 3.** Influence of vital *B. subtilis* DSM 32315 (BS) and *B. velezensis* CECT 5940 (BV) on the expression of the transforming growth factor β (TGF-β) genes (A) *TGFB1* and (B) *TGFB2* in chicken PBMCs**.** Data represent normalized read counts of six biological replicates after treatment with BS or BV. Results are presented as scatter dot plots showing the mean with SD. Individual values represent the mean of the technical replicates per biological replicate and are shown as circles (Control), squares (BS), and triangles (BV). Same colour refers to the same individual for control and BS or BV treatment. A paired t-test (BS: *TGFB1*; BV: *TGFB1*, *TGFB2*) or a Wilcoxon rank-sum test (BS: *TGFB2*) was performed. Significance is shown as +, p < 0.1; *, p < 0.05.


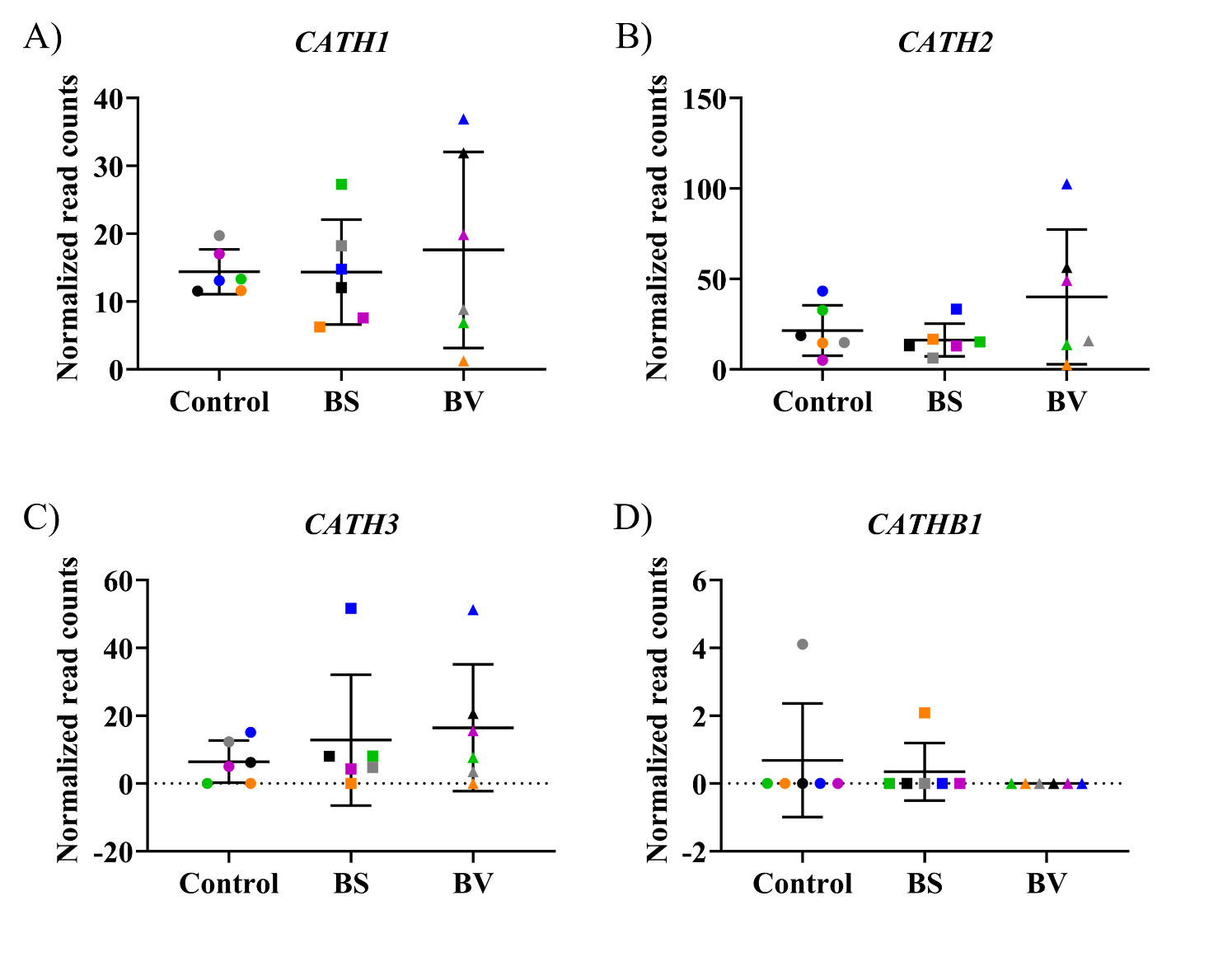


**Supplementary Figure 4.** Expression of cathelicidin genes (*CATH1*, *CATH2*, *CATH3*, *CATHB1*) in chicken PBMCs after treatment with vital *B. subtilis* DSM 32315 (BS) and *B. velezensis* CECT 5940 (BV). Expression levels of (A) *CATH1*, (B) *CATH2*, (C) *CATH3*, and (D) *CATHB1*. Data represent normalized read counts of six biological replicates after treatment with BS or BV. Results are presented as scatter dot plots showing the mean with SD. Individual values represent the mean of the technical replicates per biological replicate and are shown as circles (Control), squares (BS), and triangles (BV). The same colour refers to the same individual for control and BS or BV treatment. A paired t-test (BS: *CATH1*, *CATH2*; BV: *CATH1*, *CATH2*, *CATH3*) or a Wilcoxon rank-sum test (BS: *CATH3*, *CATHB1*; BV: *CATHB1*) was performed.
